# Leveraging *in vitro* and pharmacokinetic models to support bench to bedside investigation of XTMAB-16 as a novel pulmonary sarcoidosis treatment

**DOI:** 10.3389/fphar.2023.1066454

**Published:** 2023-03-20

**Authors:** Elliot Offman, Noopur Singh, Mark W. Julian, Landon W. Locke, Sabahattin Bicer, Jonah Mitchell, Thomas Matthews, Kirsten Anderson, Elliott D. Crouser

**Affiliations:** ^1^ Certara, Princeton, NJ, United States; ^2^ Xentria, Inc., Chicago, IL, United States; ^3^ Division of Pulmonary, Critical Care and Sleep Medicine, The Dorothy M. Davis Heart and Lung Research Institute, Columbus, OH, United States; ^4^ Department of Microbial Infection and Immunity, The Ohio State University Wexner Medical Center, Columbus, OH, United States; ^5^ Department of Biomedical Engineering, The Ohio State University, Columbus, OH, United States

**Keywords:** sarcoidosis, XTMAB-16, TNFα, granuloma, population pharmacokinetic modeling

## Abstract

**Background:** Sarcoidosis is a chronic, multisystem inflammatory disorder characterized by non-caseating epithelioid granulomas; infiltration of mononuclear cells; and destruction of microarchitecture in the skin, eye, heart, and central nervous system, and the lung in >90% of cases. XTMAB-16 is a chimeric anti-tumor necrosis factor alpha (TNFα) antibody, distinct from other anti-TNF antibodies based on its molecular structure. The efficacy of XTMAB-16 has not been clinically demonstrated, and it is still undergoing clinical development as a potential treatment for sarcoidosis. The current study demonstrates the activity of XTMAB-16 in a well-established *in vitro* sarcoidosis granuloma model, although XTMAB-16 is not yet approved by the United States Food and Drug Administration (FDA) for treatment of sarcoidosis, or any other disease.

**Objective:** To provide data to guide safe and efficacious dose selection for the ongoing clinical development of XTMAB-16 as a potential treatment for sarcoidosis.

**Methods:** First, XTMAB-16 activity was evaluated in an established *in vitro* model of granuloma formation using peripheral blood mononuclear cells from patients with active pulmonary sarcoidosis to determine a potentially efficacious dose range. Second, data obtained from the first-in-human study of XTMAB-16 (NCT04971395) were used to develop a population pharmacokinetic (PPK) model to characterize the pharmacokinetics (PK) of XTMAB-16. Model simulations were performed to evaluate the sources of PK variability and to predict interstitial lung exposure based on concentrations in the *in vitro* granuloma model.

**Results:** XTMAB-16 dose levels of 2 and 4 mg/kg, once every 2 weeks (Q2W) or once every 4 weeks (Q4W) for up to 12 weeks, were supported by data from the non-clinical, *in vitro* secondary pharmacology; the Phase 1 clinical study; and the PPK model developed to guide dose level and frequency assumptions. XTMAB-16 inhibited granuloma formation and suppressed interleukin-1β (IL-1β) secretion in the *in vitro* granuloma model with a half maximal inhibitory concentration (IC_50_) of 5.2 and 3.5 μg/mL, respectively. Interstitial lung concentrations on average, following 2 or 4 mg/kg administered Q2W or Q4W, are anticipated to exceed the *in vitro* IC_50_ concentrations.

**Conclusion:** The data presented in this report provide a rationale for dose selection and support the continued clinical development of XTMAB-16 for patients with pulmonary sarcoidosis.

## Introduction

Sarcoidosis is a chronic, multisystem inflammatory disorder of unknown etiology that is characterized by the presence of non-caseating epithelioid granulomas, accompanied by infiltration of mononuclear cells and destruction of microarchitecture. This rare disease affects the skin, eye, heart, and central nervous system, and >90% of cases involve the lungs. Patients with lung manifestations may progress to loss of lung function, and immune-modulating therapy may be beneficial in these cases (Callejas-Rubio et al., 2008). Current treatment options are suboptimal due to adverse side effects, limited efficacy, or inability to access the most effective treatments (Gerke, 2020). First-line agents include corticosteroids (tapered over time), followed by second- and third-line immunomodulatory agents, which are carefully selected to mitigate side effects, particularly related to systemic steroid exposure. There is a clear need for targeted treatment options for people living with sarcoidosis.

Macrophage-derived tumor necrosis factor alpha (TNFα) participates in the induction and maintenance of granulomas, and high levels of TNFα (released from alveolar macrophages) seem to correlate with disease progression (Callejas-Rubio et al., 2008; Crouser et al., 2017). In chronic sarcoidosis, prevention of granuloma formation and limitation of tissue injury and fibrosis are the goals of therapy. In some cases, these can be achieved through targeted immunosuppression of pro-inflammatory cytokines such as TNFα. Infliximab is a monoclonal antibody (mAb) targeting TNFα that is being used off-label to treat sarcoidosis. In a randomized controlled study of patients with chronic refractory pulmonary sarcoidosis, low- and high-dose intravenous (IV) infusion of infliximab produced a 2.5% increase in predicted forced vital capacity at 24 weeks (Baughman et al., 2006). Notably, other TNF inhibitors have been shown to be ineffective for the treatment of sarcoidosis (Judson et al., 2014) or can paradoxically cause sarcoidosis-like drug reactions (Chopra et al., 2018), suggesting that further study of the use of TNF inhibitors to treat sarcoidosis is needed.

XTMAB-16 is a chimeric human-murine immunoglobulin G 1 kappa (IgG 1κ) anti-TNFα antibody with a molecular weight of ∼149 kDa. XTMAB-16 consists of a 25% mouse variable region and a 75% human constant region. XTMAB-16 is distinct from other anti-TNF antibodies (e.g., adalimumab, infliximab) based on its molecular structure. These structural differences may potentially mitigate safety and immunogenicity concerns with this type of antibody. XTMAB-16 was developed using a Chinese Hamster Ovary cell line rather than an SP2/0 cell line (An et al., 2019). The main sialic acid form in XTMAB-16 is N-acetylneuraminic acid (Neu5Ac), the predominant sialic acid found in human cells. Whereas the main sialic acid form of infliximab is N-glycolylneuraminic acid (Neu5Gc) (Ghaderi et al., 2012). Neu5Gc is not produced in human cells and has been demonstrated to be immunogenic (Altman and Gagneux, 2019). Therefore, the lack of Neu5Gc in XTMAB-16 along with the use of alternative expression systems may be considered important structural differences that may potentially mitigate safety and immunogenicity concerns with this type of antibody.

The safety, tolerability, immunogenicity, and pharmacokinetics (PK) of XTMAB-16 have been characterized in normal healthy volunteers in a first-in-human study (NCT04971395). While the efficacy of XTMAB-16 has not been clinically demonstrated, in the current studies, we used a well-established *in vitro* sarcoidosis granuloma model (Crouser et al., 2017; Locke et al., 2019; Locke et al., 2020; Crouser et al., 2021; Drent et al., 2021; Bhargava et al., 2022) to compare XTMAB-16 suppression of sarcoidosis granuloma formation to suppression with glucocorticoids. Based on *in vitro* concentration responses and PK data obtained in humans from a first-in-human study, modeling and simulations were performed to inform the optimal clinical paradigm predicted to achieve therapeutically relevant lung tissue drug concentrations.

## Materials and methods

### *In vitro* granuloma model of sarcoidosis

The *in vitro* sarcoidosis model of granuloma formation has been described previously and is validated to closely replicate the molecular features of granulomas in diseased sarcoidosis tissues (Crouser et al., 2017; Locke et al., 2019; Locke et al., 2020; Crouser et al., 2021; Drent et al., 2021; Bhargava et al., 2022). Briefly, peripheral blood mononuclear cells (PBMCs) from patients with active pulmonary sarcoidosis with lung and/or lymph node involvement who were naïve to tuberculosis (TB) (negative purified protein derivative [PPD] skin test and/or Quantiferon Gold test; *n* = 10), non-smokers, and not treated with potent immune suppressants (e.g., methotrexate, azathioprine, prednisone, anti-TNF antibodies) within 3 months, were isolated and cultured in standard 24-well plates. PMBCs were exposed to either uncoated beads (UNC) or *mycobacterium tuberculosis* (*M. tb*) antigen PPD-coated beads for 7 days. Sarcoid PBMCs were cultured in the presence or absence of inhibitor pre-treatments XTMAB-16 or prednisone (in a dose-range demonstrated to suppress cultured human PBMC inflammatory responses *in vitro* in prior studies (Fitzgerald et al., 2004)) for 30 min followed by exposure to the PPD-coated beads (Supplementary Figure S1). Images of the cells at 7 days post-treatment were acquired using a Leica Stellaris 5 confocal microscope (Leica Microsystems, Deerfield, IL, United States) using the 10x objective with a magnification of 1. During imaging, the well plate was placed in a 37°C environmental control chamber (H201 T Unit-BL with 5% CO_2_/air perfusion; Oko Lab, Ambridge, PA, United States). Quantitative analysis of granuloma size/frequency was performed using MIPAR image-processing software. Area fraction, defined as the cumulative granuloma area as a percentage of the total area of the image, was calculated for each treatment group based on at least four independent images. Following imaging, supernatant samples were collected and quantified for extracellular cytokine release using an enzyme-linked immunosorbent assay.

Granuloma formation following pre-treatment with XTMAB-16 (1, 5, 10, 20, and 40 μg/mL) or prednisone (0.358 and 3.58 μg/mL) (30 min prior to PPD-coated bead exposure) was compared to that following treatment of PPD-coated beads alone. UNC served as comparable negative controls.

The *in vitro* study was conducted in accordance with the amended Declaration of Helsinki, and donors were enrolled after first obtaining informed, written consent in compliance with The Ohio State University Biomedical Sciences Institutional Review Board (#2014H0380) and National Institutes of Health guidelines. In addition, the *in vitro* model studies are under the ClinicalTrials.gov identifier, NCT01857401.

### Phase 1 XTMAB-16 study

XTMAB-16-101 (NCT04971395) was a randomized, double-blind, placebo-controlled, first-in-human study to evaluate the safety, tolerability, PK, and immunogenicity profile of single IV infusions of XTMAB-16 in healthy adults. None of the patients recruited for this Phase 1 study were smokers. XTMAB-16 was administered *via* IV infusion over a 2-h period at doses of 2 or 4 mg/kg. Based on the established no-observed-adverse-effect level (NOAEL) of XTMAB-16 in the non-clinical program (40 mg/kg), the maximum clinical starting dose was 4 mg/kg.

Inclusion criteria included healthy adult male or female; aged 18–45 years, inclusive; with a weight of between 45 and 100 kg and a body mass index (BMI) between 18.0 and 30.0 kg/m^2^, inclusive; and with clinically acceptable clinical laboratory values and electrocardiogram results. Exclusion criteria included receipt of any investigational compound within 90 days prior to dosing and any significant illness. A total of 25 normal healthy adult participants were enrolled and assigned into two treatment cohorts (2 or 4 mg/kg) or placebo. The mean age (min, max) of the participants was 32 years (19, 45); the majority were female (*n* = 16, 64%), Black or African American (*n* = 20, 80%), and not Hispanic or Latino (*n* = 19, 76%); with a mean BMI (min, max) of 25.84 (20.9, 29.8) (Supplementary Table S1, Supplementary Methods).

### Pharmacokinetic analysis

#### Data

The PK analysis was performed on data collected in Study XTMAB-16-101 (NCT04971395). For the population PK (PPK) analysis, the dataset included information on subject identifier, date and time of XTMAB-16 dosing, nominal and actual date and time of PK sample collections, concentrations of XTMAB-16 in serum, as well as data of relevant covariates, including body weight, age, height, BMI, dose, subject-level anti-drug antibody (ADA), sample-level ADA, sex, race, and ethnicity. For the purposes of this analysis, below the limit of quantitation (BLQ) samples were set to missing.

#### PPK model development

PPK model development was performed using Phoenix^®^ NLME (v 8.4, Certara). A two-compartment model was used as the starting point for this modeling exercise based on visual inspection of concentration-time profiles.

#### Covariate analysis

Following the selection of a base PPK model, the relationships between covariates and PK parameters of XTMAB-16 were first explored graphically to obtain information on covariates likely to affect the parameters of interest, and to guide the covariate analysis. Correlations between covariates were considered during the covariate selection process (i.e., where two covariates were assumed or demonstrated graphically to correlate, only one of the two would be selected in a formal covariate model testing strategy).

#### Model assessment

Model evaluation was based on standard model diagnostics and goodness-of-fit criteria (e.g., accuracy of parameter estimation [i.e., 95% confidence interval excluding 0], successful model convergence) and by looking at pertinent graphical representations of goodness-of-fit (e.g., fitted and observed concentrations *versus* time, conditionally weighted residuals *versus* time).

An internal qualification of the final model was performed through a prediction-corrected visual predictive check (pcVPC). A pcVPC was constructed using the final model estimates and involved simulating 1,000 replicates of concentration-time profiles, followed by normalizing based on the typical population prediction to help account for differences in study design.

Following a successful evaluation of the final PPK model, individual estimates of PK and exposure parameters were derived by posterior Bayesian estimation and compared with PK parameters previously determined by non-compartmental analysis.

#### Simulations

Biweekly (Q2W) and once every 4-week (Q4W) regimens were simulated at 2 and 4 mg/kg over the course of 16 weeks. A total of six doses were administered in the Q2W regimen and three doses were administered in the Q4W regimen in the first 12 weeks. Monte-Carlo simulations were performed whereby each virtual subject’s (*N* = 500) PPK model parameters were obtained from sampling *via* the final PPK variance-covariance matrix. To account for body weight in the simulations, body weight was assigned randomly from a uniform distribution of 50–100 kg. The infusion duration of XTMAB-16 was assumed to be 2 h for each virtual subject. The ADA status was included as a predictor in the simulation.

To account for concentrations in lung tissue, interstitial lung concentration of XTMAB-16 was estimated using the antibody biodistribution coefficient for lung tissue (14.9%) (Li et al., 2016). Exposure parameters for a typical individual weighing 70 kg and with an ADA negative status following the first dose and the last dose in the lung and in serum were then calculated and presented in tabular format for illustrative purposes.

## Results

### Activity of XTMAB-16 in the *in vitro* Granuloma model of sarcoidosis

Demographic characteristics of the patients included in the *in vitro* study are shown in Supplementary Table S2. The designation of high or low responder indicates the response to PPD-coated beads in terms of *in vitro* granuloma formation (Crouser et al., 2017).

Representative photomicrographs of granuloma-like cell aggregates forming 7 days after high responder sarcoidosis PBMCs were incubated with either UNC or PPD-coated beads following XTMAB-16 or prednisone pre-treatment are shown in Supplementary Figure S2. The data show that XTMAB-16 pre-treatment significantly attenuates granuloma formation in the *in vitro* model in a dose-dependent fashion within the range of 1–40 μg/mL. The effect of XTMAB-16 at the higher doses in the *in vitro* granuloma model was comparable to that of prednisone (0.358–3.58 μg/mL) pre-treatment.

**FIGURE 1:**
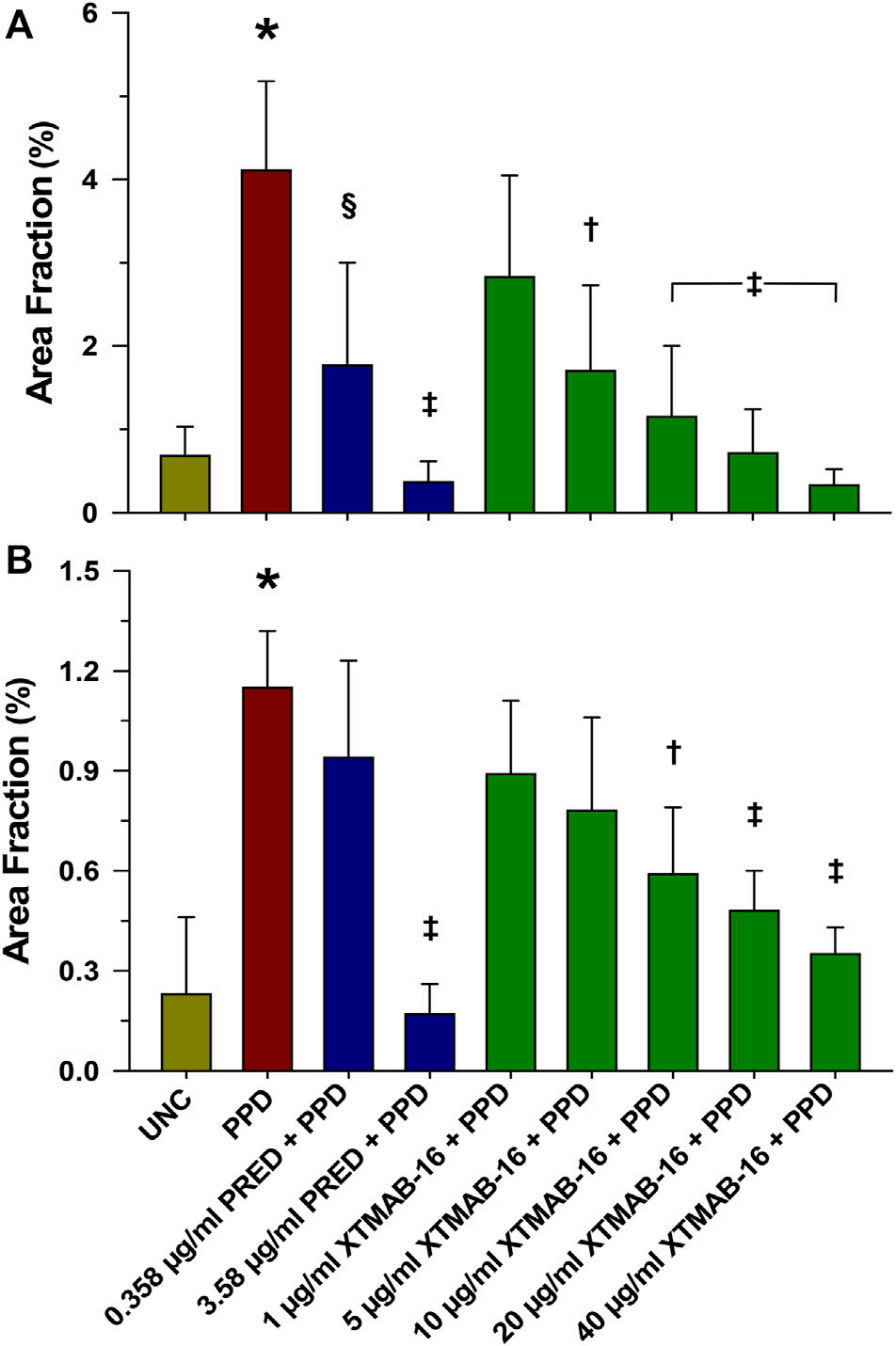
Group Analysis of Granuloma Formation Represented as Area Fraction in High Responder **(A)**, and Low Responder **(B)** Patients With Sarcoidosis. Note: *n* = 6 for the high responder group, and *n* = 4 for the low responder group; *p < 0.001, compared to the uncoated bead (UNC) treatment group; †*p* < 0.01, ‡*p* < 0.001, and §*p* < 0.05 relative to the PPD-treated group. Area Fraction = cumulative granuloma area as a percentage of the total area of the image. Bars are mean (percent) ± SD. Abbreviations: PPD = purified protein derivative; PRED = prednisone; SD = standard deviation; UNC = uncoated beads.

XTMAB-16 pre-treatment for 30 min had a significant dose-response effect in the *in vitro* granuloma model to suppress granuloma formation 7 days following treatment with PPD as calculated by the area fraction (Figure 1). For comparison, similar responses to prednisone pre-treatments were demonstrated.

**FIGURE 2:**
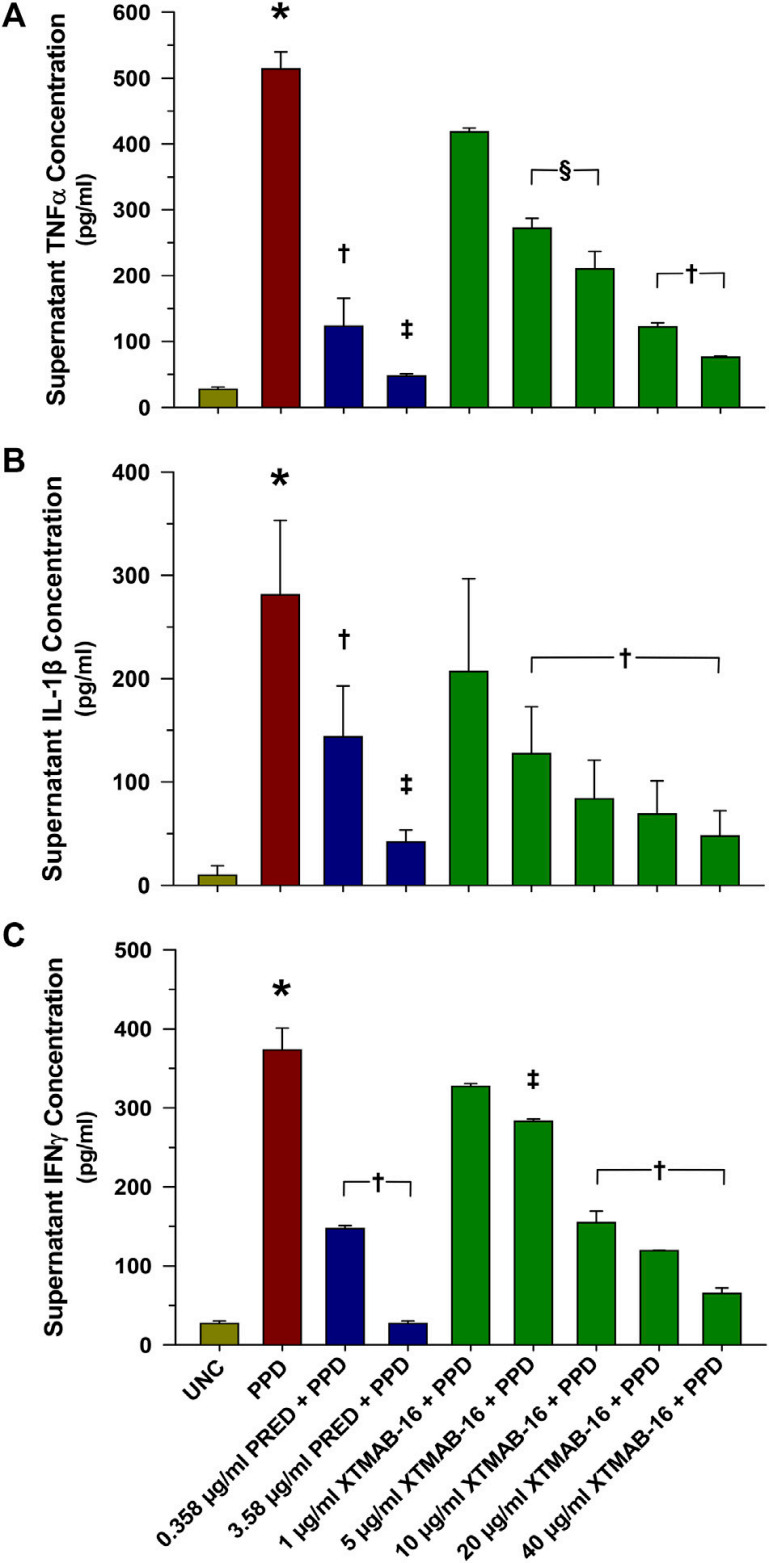
Group Supernatant TNFα **(A)**, IL-1β **(B)**, and IFNγ **(C)** Release in High Responder Patients (as Defined by Having >2% Granuloma Area Fraction) With Sarcoidosis. Note: *n* = 6 for each group; *p < 0.001, compared to the uncoated bead (UNC) treatment group; †*p* < 0.01, ‡*p* < 0.001 (*p* < 0.05 in bottom), and §*p* < 0.05 relative to the PPD-treated group. Bars are mean (μg/mL)±SD. Abbreviations: IFN = interferon; IL = interleukin; PPD = purified protein derivative; PRED = prednisone; SD = standard deviation; TNF = tumor necrosis factor; UNC = uncoated beads.

XTMAB-16 pre-treatment for 30 min had a significant dose-response effect in the *in vitro* granuloma model to suppress granuloma cytokine release 7 days following treatment with PPD (Figure 2). For comparison, similar responses to prednisone pre-treatments were demonstrated.

**FIGURE 3:**
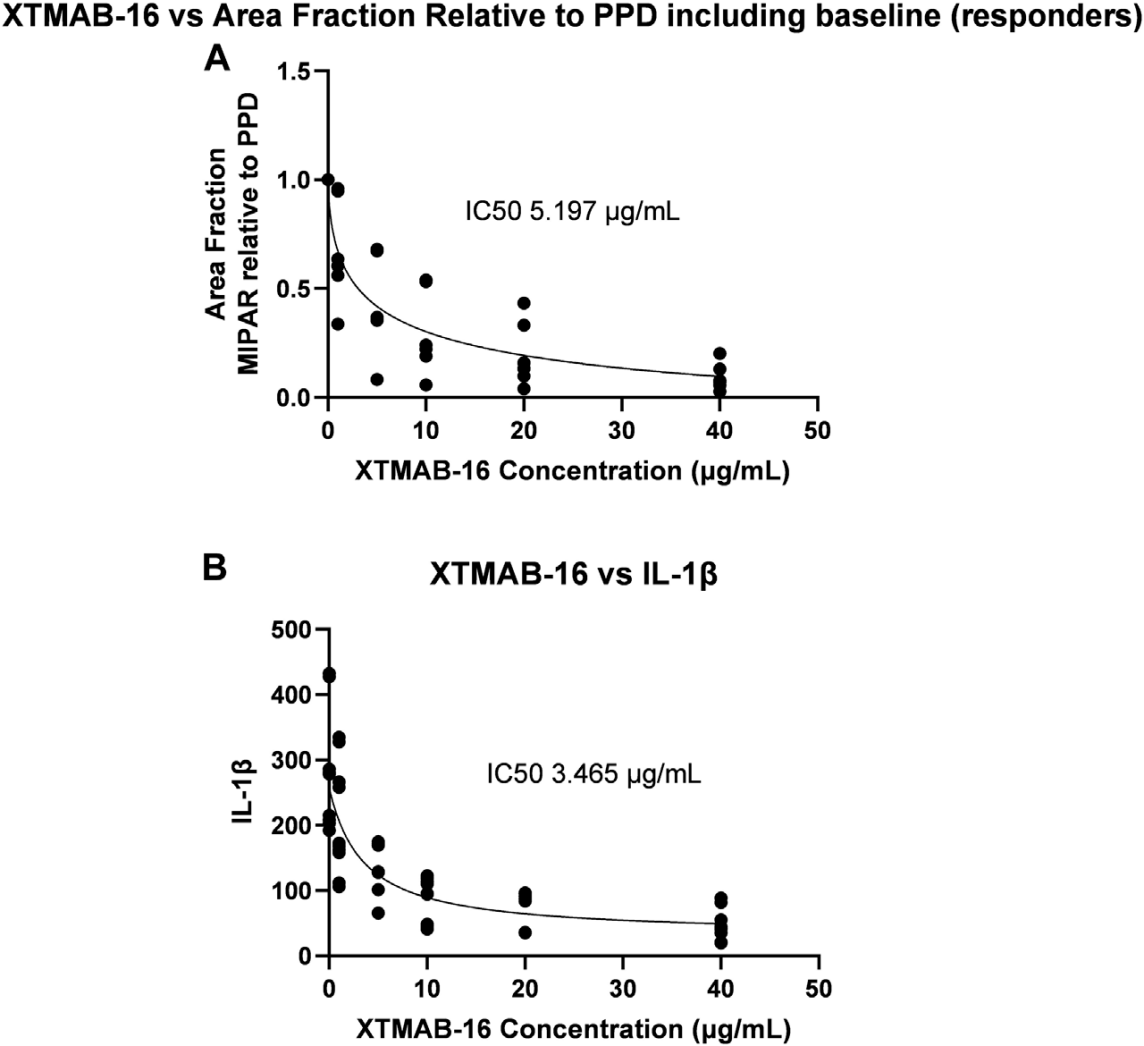
XTMAB-16 Concentration Versus Area Fraction **(A)** and IL-1β **(B)** in the *In Vitro* Granuloma Model. Note: The 95% CI was not estimated because the software was unable to calculate a complete CI. Abbreviations: CI = confidence interval; IC_50_ = half maximal inhibitor concentration; IL = interleukin; PPD = purified protein derivative.

A separate statistical analysis was performed whereby all subject samples were evaluated for XTMAB-16 half maximal inhibitory concentration (IC_50_) against the MIPAR area fraction and the interleukin (IL)-1β concentration in the *in vitro* granuloma assay supernatant (Figure 3). The 95% confidence interval (CI) for the IC_50_ was not estimated because the software was unable to calculate a complete confidence interval, and therefore the IC_50_ best-fit should be interpreted with caution. The IC_50_ for decrease in area fraction was 5.197 μg/mL and for decrease in IL-1β was 3.465 μg/mL.

In summary, *in vitro* granuloma responses varied from one patient to the next. Despite this variability, there was a consistent dose-response to XTMAB-16 *in vitro*, reflected by objective imaging to assess granuloma area. Results are comparable to prednisone. IL-1β (and not TNFα or IFNy) best reflects granuloma response in this *in vitro* model and, therefore, may be a useful biomarker for future clinical studies in sarcoidosis (Prior et al., 1996; Mikuniya et al., 2000). Based on the *in vitro* dose-response to XTMB-16, PPK modeling and simulation were performed using first-in-human study data to inform ongoing clinical development of XTMAB-16 for the treatment of sarcoidosis.

### PPK modeling

#### PK measurements

The PK analysis dataset included 277 PK observations from 19 subjects. Measurable PK observations were 226 (81.6%); pre-dose BLQ samples were 19 (6.9%) and post-treatment BLQ samples were 32 (12.4%). BLQ observations after administration of the first dose (“post-treatment BLQ”) account for 12.4% of all post-dose observations. No serum XTMAB-16 samples with a quantifiable/reported concentration value were excluded from the analysis. No individual subjects were excluded from the analysis.

#### Participant characteristics

Baseline values of continuous and categorical covariates are summarized in Table 1. Descriptive statistics of ADA samples are presented in Supplementary Table S3.

**TABLE 1:** Summary of continuous and categorical covariates.

Covariate	Study XTMAB16-101 (N = 19)
Continuous Covariates, mean (SD); median [min, max]	
Weight (kg)	72.3 (11.7); 72.4 [53.2, 97.2]
Age (years)	31.8 (6.2); 32 [19, 45]
BMI (kg/m^2^)	25.7 (3.0); 25.9 [20.9, 29.8]
Height (cm)	167.5 (9.6); 163 [158, 185]
Categorical Covariates, n (%)	
Sex, male	6 (31.6)
Caucasian race	2 (10.5)
Black or African American	16 (84.2)
American Indian or Alaska Native	1 (5.3)
ADA positive	8 (42.1)
ADA negative	11 (57.9)
Subject level neutralizing ADA positive	8 (42.1)
Subject level neutralizing ADA negative	11 (57.9)

Note: A subject with at least one positive ADA, sample is considered to have a positive ADA, status. Abbreviations: ADA, anti-drug antibody; BMI, body mass index; max, maximum; min, minimum; N, number of subjects with available information; n, number of participants in specific category; SD, standard deviation.

#### PPK analysis

**FIGURE 4A:**
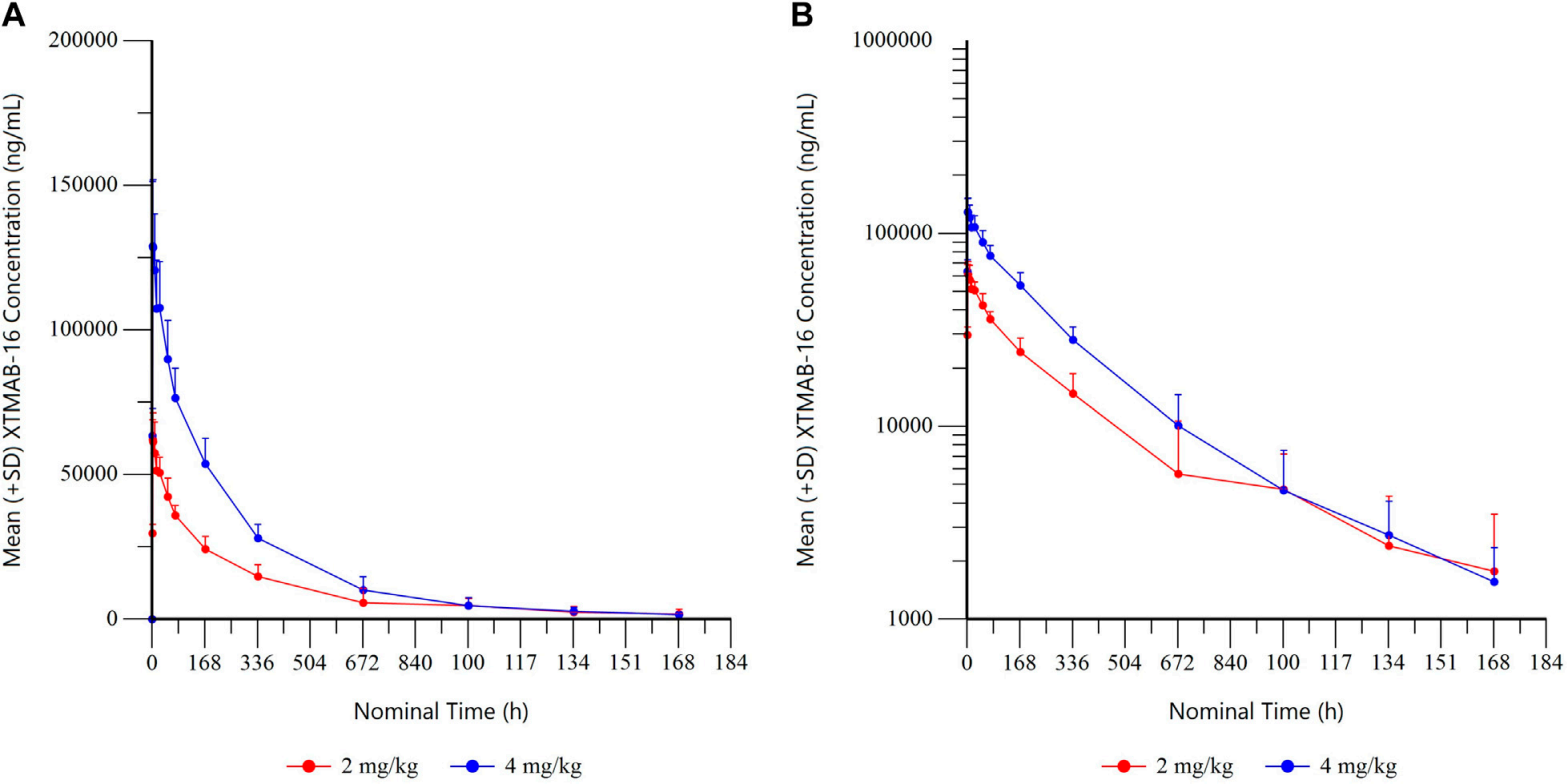
Summary XTMAB-16 Concentration Versus Time by Dose. **(A)** linear, **(B)** semi-log. Note: All BLQ samples after T_max_ were set to missing for calculation of summary statistics. Abbreviations: BLQ = below the limit of quantitation; SD = standard deviation; T_max_ = time to maximum concentration.

The arithmetic mean (± standard deviation) serum drug concentration-time profiles of the study population are shown in Figure 4. The shape of the elimination phase in the semi-log plots suggests two-compartment kinetics, while the XTMAB-16 concentration *versus* time profiles suggest a dose-proportional exposure with increasing doses.

##### Model development and qualification

A two-compartment model was considered suitable as an initial structural model. Inter-individual variability (IIV) was evaluated on all parameters. However, due to the lack of improvement with IIV on inter-compartmental clearance (Q), only IIV on clearance (CL), central volume of distribution (V), and peripheral volume of distribution (V2) were supported and retained in the model. A combined additive and proportional error model was selected to describe the residual error in the model. Weight and subject-level ADA were found to be significant influences on the PK of XTMAB-16. The parameter estimates for the final PPK model are presented in Supplementary Table S4. Based on the final model, the CL is 0.009 L/h, the volume of distribution at steady state is 3.02 L, and the estimated half-life for a typical individual (with a weight of 72.4 kg) is 243.3 h (194.0 h if subject is ADA positive). Parameters of the final XTMAB-16 PPK model were estimated with good precision; the percentages of the relative standard error for PK parameters and the random effects were <23%.

The final model resolved residual covariate trends with respect to body weight and sex on the central volume of distribution; however, a small residual trend in the peripheral volume remained with respect to sex. Addition of a covariate on sex, however, did not result in a significant drop in objective function value (Δ-2LL = 5.01), and thus the sex covariate was not included in the final model. Due to small sample sizes, the impact of race and ethnicity could not be robustly estimated.

Goodness-of-fit plots also suggest reasonable fit of the data (Supplementary Figure S3). Individual- and population-predicted concentrations showed good correlation with observed data. The conditional weighted residuals (CWRES) were evenly distributed around 0 and showed no trends over time or by concentration, thus indicating a lack of bias in the model.

A pcVPC was performed to qualify the model, whereby 1,000 replicates of the observed subjects were simulated. A pcVPC plot (linear-log) for the final XTMAB-16 PK model concentrations is presented in Supplementary Figure S4. The final PPK model was able to predict the observed median and 5th and 95th percentiles of observed XTMAB-16 concentrations with good accuracy. Model evaluation by pcVPC showed suitable predictive performance of the model, which captured both the central tendency and variability in observed concentrations.

##### Simulations

Simulated XMTAB-16 serum and lung exposure increased with increasing doses. For a given mg/kg dose, the Q2W dosing regimen produced a higher trough concentration (C_trough_) and average concentration (C_avg_) compared to the Q4W dosing regimen (Supplementary Tables S5, S6). PK parameters for simulated serum XTMAB-16 concentrations following single administration are shown in Supplementary Table S7.

**FIGURE 5:**
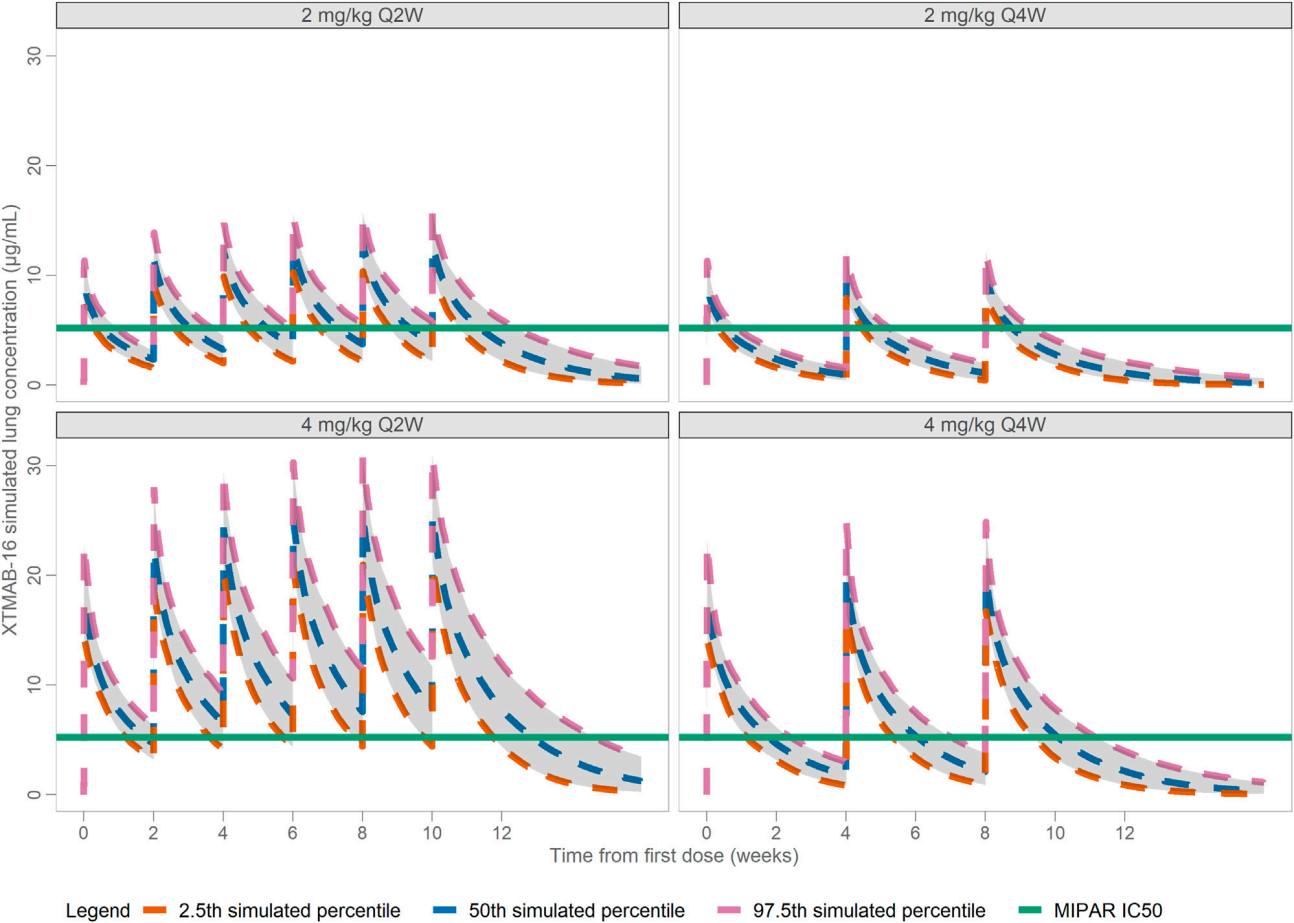
Model-Predicted Lung XTMAB-16 Concentration in Virtual Subjects. Abbreviations: IC_50_ = concentration at half-maximal MIPAR area fraction defined as the cumulative granuloma area as a percentage of the total area of the image; Q2W = every 2 weeks; Q4W = every 4 weeks.

After accounting for the anticipated biodistribution for an mAb from plasma to lung tissue, simulations in adult subjects suggested that 4 mg/kg Q2W would largely achieve C_trough_ values in lung that exceed the IC_50_ that demonstrated reduction in granuloma growth in the *in vitro* sarcoidosis model (granuloma area fraction IC_50_ = 5.197 μg/mL; IL-1β IC_50_ = 3.465 μg/mL); whereas doses of 2 mg/kg Q2W or Q4W or 4 mg/kg Q4W would fall below the *in vitro* IC_50_ (Figure 5). The C_avg_ predicted lung exposure is similar to the granuloma IC_50_ for the 4 mg/kg Q2W regimen with the remaining regimens falling below the IC_50_. Thus, the proposed clinical dose regimens are predicted to provide for a range of XTMAB-16 PK lung exposure in the steep portion of the exposure-response curve.

## Discussion

There is a demonstrated need for targeted treatment options for people living with sarcoidosis. Sarcoidosis granulomas are distinct from other granuloma types, and this has implications for treatment. For instance, a prior study utilizing this *in vitro* model (Crouser et al., 2017) indicated that TNFα production is higher in sarcoidosis granulomas formed by PBMCs in response to PPD-coated beads compared to identically treated PBMCs from disease-free controls or latent TB treated with PPD-coated beads. Anti-TNF therapy has been shown to improve lung function in a subset of sarcoidosis patients with active nodular lung inflammation on X-ray (Baughman et al., 2006; Vorselaars et al., 2015).

XTMAB-16 is a chimeric anti-TNFα antibody with structural differences compared to other anti-TNF antibodies including the lack of Neu5Gc and the use of an alternative expression system, which may mitigate safety and immunogenicity concerns with this type of antibody. XTMAB-16 safety, tolerability, immunogenicity, and PK have been characterized in normal healthy volunteers in a first-in-human study (NCT04971395). While the efficacy of XTMAB-16 has not been clinically demonstrated, the current study demonstrates the activity of XTMAB-16 in a well-established *in vitro* sarcoidosis granuloma model (Crouser et al., 2017; Locke et al., 2019; Locke et al., 2020; Crouser et al., 2021; Drent et al., 2021; Bhargava et al., 2022). In addition, a PPK analysis was developed using PK data from the XTMAB-16 first-in-human study, followed by simulations to guide dose selection for the ongoing clinical development of XTMAB-16 for the treatment of sarcoidosis.

Although responses may vary in the *in vitro* model, there was a consistent dose-response to XTMAB-16 *in vitro*, as reflected by objective imaging to assess granuloma area. It is hypothesized that this variability may reflect disease activity (e.g., based on unpublished data showing reduced granuloma responses when sarcoidosis patients are receiving potent immune modulating agents, which is the basis for excluding such subjects from participation in this study). XTMAB-16’s granuloma-suppressing activity was comparable to prednisone, the established first-line therapy for this condition (Rahaghi et al., 2020).

In addition, IL-1β, linked to sarcoidosis in a number of recent publications (Talreja et al., 2019; Huppertz et al., 2020; Wahlund et al., 2020), appears to be a reliable predictor of granuloma response in this model and, therefore, could act as a biomarker for response in future studies. The reason TNFα may be inferior to IL-1β at reflecting granuloma formation is unclear, but TNFα levels in sarcoidosis tissues are known to vary greatly based, in part, on disease activity (Lepzien et al., 2021).

The PK of XTMAB-16 following IV administration was well characterized by a two-compartment model. A covariate search determined weight on CL, V, and V2, and subject-level ADA on CL were significant covariates. There was insufficient sample size to robustly evaluate the contribution of ethnicity or race on the PK of XTMAB-16; however, PK parameters were largely overlapping for subjects noted as Hispanic/Latino compared to those noted as non-Hispanic/non-Latino. There were no trends detected with respect to age, and minimal residual trends with respect to sex, on the PK of XTMAB-16. XTMAB-16 was only evaluated in this current analysis following single doses, and the impact of positive ADA status on repeated dosing cannot be assumed to be consistent with the clinical scenario in patients receiving immunosuppressive therapy, therefore, only simulations in ADA-negative patients are presented.

Limitations of this study include the inter-sample variation in disease activity based on the *in vitro* granuloma model and also based on well-established disease phenotypes such as Lӧfgren’s (self-limited disease) and non-Lӧfgren’s (progressive) and related TNFα responses (Lepzien et al., 2021). Another limitation is the use of healthy participants and not patients with sarcoidosis in the Phase 1 study, given that patients on immunosuppressive therapies would likely have a different ADA response. Therefore, the PK simulations may not completely represent sarcoidosis patients. In addition, an assumption was made that the effective trough and C_avg_ were represented by the interstitial fluid based on antibody biodistribution coefficients, and drug permeability into a granuloma may be less or more permeable and patients may warrant higher systemic exposure. However, exposure of other anti-TNF mAbs was considered and showed trough values within the range of the expected steady-state C_trough_ anticipated for the XTMAB-16 clinical regimens (Crommelin, 2018).

The PPK modeling analysis presented here was utilized to guide safe and potentially efficacious dose selection for ongoing XTMAB-16 clinical development as a potential treatment for sarcoidosis. PPK model simulations will continue to be leveraged throughout the clinical development of XTMAB-16 to explore potential racial and ethnic PK differences and to help manage risk and accelerate the potential of a new treatment to patients living with sarcoidosis.

In summary, XTMAB-16 dose levels of 2 and 4 mg/kg, Q2W or Q4W for up to 12 weeks are supported by data from the non-clinical, *in vitro* secondary pharmacology; the Phase 1 clinical study; and the PPK model developed to guide dose level and frequency assumptions. Predicted C_avg_ and C_trough_ of XTMAB-16 in lung tissue approximate the IC_50_ values for reduction in granuloma formation and IL-1β in the *in vitro* granuloma model. Utilization of this well-established *in vitro* model of sarcoidosis enables continued testing of model assumptions as clinical data become available. The data presented provide a rationale for dose selection and support the continued clinical development of XTMAB-16 for patients with pulmonary sarcoidosis.

## Data Availability

The original contributions presented in the study are included in the article/Supplementary Material, further inquiries can be directed to the corresponding author.
