# Supplementary material for: Leveraging in vitro and pharmacokinetic models to support bench to bedside investigation of XTMAB-16 as a novel pulmonary sarcoidosis treatment
Source: Front Pharmacol. 2023 Mar 20;14:1066454. doi: 10.3389/fphar.2023.1066454 (PMC10067675; doi:10.3389/fphar.2023.1066454)
Supplement: Supplementary file 1 [file DataSheet1.docx]

Elliot Offman^1*^, Noopur Singh^2^, Mark W. Julian^3^, Landon W. Locke ^4,5^, Sabahattin Bicer^5^, Jonah Mitchell^5^, Thomas Matthews^2^, Kirsten Anderson^2^, Elliott D. Crouser^3^

^1^Certara, Princeton, NJ, USA; ^2^Xentria, Inc., Chicago, IL, USA; ^3^Division of Pulmonary, Critical Care and Sleep Medicine, The Dorothy M. Davis Heart and Lung Research Institute, ^4^Department of Microbial Infection and Immunity, The Ohio State University Wexner Medical Center, and ^5^Department of Biomedical Engineering, The Ohio State University, Columbus, OH, USA

**Corresponding author:**

Elliott D. Crouser, M.D.

Division of Pulmonary, Critical Care and Sleep Medicine

The Dorothy M. Davis Heart and Lung Research Institute

The Ohio State University Wexner Medical Center, Columbus, OH, USA

Email: Elliott.Crouser@osumc.edu

Phone: 614-361-6423

Supplementary Material

# Supplementary Material

## Supplementary Methods

## Phase 1 XTMAB-16 Study

For all cohorts, 2 participants (1 assigned to XTMAB-16 and 1 assigned to placebo; sentinel cohort) were dosed at least 48 hours before the other participants in the same cohort. After blinded review of the available safety and laboratory data of the sentinel cohort by the Principal Investigator, if no notable safety signals were identified, the remaining participants within the same cohort were dosed. Participants within a cohort were to be dosed at least 1 hour apart.

The participants at the 4 mg/kg cohort received XTMAB-16 after completion of the 2 mg/kg cohort and at least 14 to 21 days of clinical assessment of the 2 mg/kg dose cohort participants.

After a screening period of up to 28 days, participants were admitted to the clinical research unit (CRU) on Day -2 for COVID-19 testing and baseline procedures. After eligibility was confirmed, participants were randomly assigned to treatment (1:1 XTMAB-16 to placebo for the sentinel cohort, 3:1 for the remaining participants) and administered study drug on Day 1. Participants remained in the CRU through Day 8 for PK, biomarkers, anti-drug antibodies (ADA), and safety assessments. Participants were discharged on Day 8 once all procedures for that day were completed. Participants returned to the CRU on Days 15, 29, 43, 57, and 71.

Safety assessments included adverse events, safety laboratory assessments, vital signs, electrocardiogram, and physical examination. PK collection and analysis were also completed in a similar manner during the course of the study.

## Tables

Table S1. Demographic Characteristics: Safety Set

| Parameters | Statistics | Cohort 1 XTMAB-16 2 mg/kg (N=10) | Cohort 2 XTMAB-16 4 mg/kg (N=9) | Pooled Placebo (N=6) | Overall (N=25) |
| --- | --- | --- | --- | --- | --- |
| Age (Years) | n | 10 | 9 | 6 | 25 |
|  | Mean | 33.0 | 30.6 | 31.7 | 31.8 |
|  | SD | 5.64 | 6.86 | 3.44 | 5.59 |
|  | Median | 32.0 | 30.0 | 31.0 | 32.0 |
|  | Min, Max | 26, 45 | 19, 42 | 28, 36 | 19, 45 |
| Sex, n (%) | Male | 3 (30.0) | 3 (33.3) | 3 (50.0) | 9 (36.0) |
|  | Female | 7 (70.0) | 6 (66.7) | 3 (50.0) | 16 (64.0) |
| Race, n (%) | American Indian or Alaska Native | 1 (10.0) | 0 (0.0) | 0 (0.0) | 1 (4.0) |
|  | Asian | 0 (0.0) | 0 (0.0) | 0 (0.0) | 0 (0.0) |
|  | Black or African American | 8 (80.0) | 8 (88.9) | 4 (66.7) | 20 (80.0) |
|  | Native Hawaiian or Other Pacific Islander | 0 (0.0) | 0 (0.0) | 0 (0.0) | 0 (0.0) |
|  | White | 1 (10.0) | 1 (11.1) | 2 (33.3) | 4 (16.0) |
|  | Other | 0 (0.0) | 0 (0.0) | 0 (0.0) | 0 (0.0) |
| Ethnicity, n (%) | Hispanic or Latino | 2 (20.0) | 2 (22.2) | 2 (33.3) | 6 (24.0) |
|  | Not Hispanic or Latino | 8 (80.0) | 7 (77.8) | 4 (66.7) | 19 (76.0) |
|  | Unknown | 0 (0.0) | 0 (0.0) | 0 (0.0) | 0 (0.0) |
| Height (cm) | n | 10 | 9 | 6 | 25 |
|  | Mean | 167.1 | 167.9 | 170.2 | 168.1 |
|  | SD | 10.42 | 9.12 | 8.84 | 9.28 |
|  | Median | 162.0 | 165.0 | 167.5 | 165.0 |
|  | Min, Max | 158, 185 | 158, 185 | 161, 186 | 158, 186 |
| Weight (kg) | n | 10 | 9 | 6 | 25 |
|  | Mean | 70.68 | 74.06 | 76.38 | 73.26 |
|  | SD | 11.783 | 12.134 | 11.566 | 11.596 |
|  | Median | 69.05 | 72.60 | 73.40 | 72.40 |
|  | Min, Max | 53.2, 97.2 | 54.3, 91.8 | 67.3, 99.0 | 53.2, 99.0 |
| Body Mass Index (kg/m^2^) | n | 10 | 9 | 6 | 25 |
|  | Mean | 25.22 | 26.22 | 26.30 | 25.84 |
|  | SD | 2.649 | 3.415 | 2.173 | 2.787 |
|  | Median | 25.45 | 27.70 | 25.90 | 25.90 |
|  | Min, Max | 21.0, 28.4 | 20.9, 29.8 | 23.8, 28.9 | 20.9, 29.8 |
| %=percentage of participants (the denominator is N)  Abbreviations: Max=maximum; Min=minimum; N=total number of participants in safety set; n=number of participants in specific category; SD=standard deviation | | | | | |

**Table S2. Demographics of Patients Included in the In Vitro Study**

| **Groups** | **Age (years)** | **Gender** | **Race** | **H/NH** | **Scadding CXR Stage** | **Extra-pulmonary** |
| --- | --- | --- | --- | --- | --- | --- |
| Sarcoidosis High Responders (n=6) | 69 | M | W | NH | III/IV | + |
|  | 61 | F | W | NH | II | - |
|  | 51 | M | W | NH | II | + |
|  | 63 | F | W | NH | II | - |
|  | 65 | F | B | NH | III/IV | + |
|  | 75 | F | W | NH | I | + |
| Mean (SEM) or Ratio | 64.0 (3.3) | 2/4 (M/F) | 5/1/0 (W/B/O) | 0/6 (H/NH) | - | - |
| Sarcoidosis Low Responders (n=5) | 61 | M | W | NH | III |  |
|  | 45 | F | B | NH | III |  |
|  | 63 | M | B | NH | IV |  |
|  | 52 | M | W | NH | I |  |
|  | 66 | F | B | NH | II |  |
| Mean (SEM) or Ratio | 57.4 (3.9) | 3/2 (M/F) | 2/3/0 (W/B/O) | 0/4 (H/NH) | - | - |
| Abbreviations: B=Black; CXR=chest X-ray; F=female; H=Hispanic; M=male; NH=non-Hispanic; O=other; SEM=standard error of the mean; W=White | | | | | | |

**Table S3. Summary of Pharmacokinetic and ADA Samples**

| **Samples** | **N (% of Total)** |
| --- | --- |
|  | **Study XTMAB16-101**  **(N=19)** |
| Total number of samples | 277 (100) |
| Total number of pre-dose samples | 19 (6.9) |
| Total number of post-dose samples | 258 (93.1) |
| Number of ADA samples | 18 (6.1) |
| Number of neutralizing positive ADA samples | 18 (6.1) |
| Abbreviations: ADA=anti-drug antibody; N=number of subjects with available information | |

**Table S4. Final Model Parameter Estimates**

| **Parameters** | **Estimates** | **%RSE** | **95% CI** |
| --- | --- | --- | --- |
| CL (L/h) | 0.00902  × 1.274 if subject is ADA positive | 6.2 | 0.008 – 0.010 |
| V (L) | 2.30  × ${(\frac{Weight}{72.4})}^{0.426}$ | 2.2 | 2.20 – 2.40 |
| V2 (L) | 0.715  × ${(\frac{Weight}{72.4})}^{0.426}$ | 13.1 | 0.53 – 0.90 |
| Q (L/h) | 0.0119 | 22.5 | 0.007 – 0.017 |
| **Random Effects^a^** | **Estimates** | **%RSE** | **Shrinkage (%)** |
| IIV on CL | 0.0246 | 6.7 | 7.1 |
| IIV on V | 0.00936 | 2.7 | 4.2 |
| IIV on V2 | 0.203 | 16.8 | 17.7 |
| **Residual Error** | **Estimates** | **%CV** | |
| Proportional error (%) | 6.89 | 9.6 | |
| Additive error (ng/mL) | 819 | 57.5 | |
| ^a^ Expressed as variance  Abbreviations: ADA=anti-drug antibody; CI=confidence interval; CL=clearance; CV=coefficient of variation; IIV=inter-individual variability; Q=inter-compartmental clearance; RSE=relative standard error; V=central volume of distribution; V2=peripheral volume of distribution | | | |

***Table S5. Simulated XTMAB-16 Exposure in Serum at Steady State (ADA Negative)***

| **Dosing**  **Regimen** | | | | **C_trough_**  **(µg/mL)** | **C_avg_**  **(µg/mL)** |
| --- | --- | --- | --- | --- | --- |
| Single Dose | Q2W | 2 mg/kg | 16.1 | - | |
|  | Q2W | 4 mg/kg | 32.3 | - | |
|  | Q4W | 2 mg/kg | 6.11 | - | |
|  | Q4W | 4 mg/kg | 12.2 | - | |
| Repeated Dose | Q2W | 2 mg/kg | 25.9 | 46.1 | |
|  | Q2W | 4 mg/kg | 51.8 | 92.1 | |
|  | Q4W | 2 mg/kg | 7.11 | 23.0 | |
|  | Q4W | 4 mg/kg | 14.2 | 46.1 | |
| Abbreviations: ADA=anti-drug antibody; C_avg_=average concentration; C_trough_=trough concentration; Q2W=every 2 weeks; Q4W=every 4 weeks | | | | | |

***Table S6. Simulated XTMAB-16 Exposure in Lung at Steady State (ADA Negative)***

| **Dosing**  **Regimen** | | | | **C_trough_**  **(µg/mL)** | **C_avg_**  **(µg/mL)** |
| --- | --- | --- | --- | --- | --- |
| Single Dose | Q2W | 2 mg/kg | 2.40 | - |  |
|  | Q2W | 4 mg/kg | 4.81 | - | |
|  | Q4W | 2 mg/kg | 0.91 | - | |
|  | Q4W | 4 mg/kg | 1.82 | - | |
| Repeated Dose | Q2W | 2 mg/kg | 3.86 | 2.40 |  |
|  | Q2W | 4 mg/kg | 7.71 | 4.81 | |
|  | Q4W | 2 mg/kg | 1.06 | 0.91 | |
|  | Q4W | 4 mg/kg | 2.12 | 1.82 | |
| Abbreviations: ADA=anti-drug antibody; C_avg_=average concentration; C_trough_=trough concentration; Q2W=every 2 weeks; Q4W=every 4 weeks | | | | | |

**Table S7. Pharmacokinetic Parameters for Simulated Serum XTMAB-16 Concentrations Following Single Administration (ADA Negative)**

|  | **C_max_**  **(µg/mL)** | **AUC_inf_**  **(h*µg/mL)** | **C_eoi_**  **(µg/mL)** | **C_trough_**  **(µg/mL)** | **Vz**  **(mL/kg)** | **Vss**  **(mL/kg)** | **CL**  **(mL/h/kg)** | **T_max_**  **(h)** | **MRT_inf_**  **(h)** | **T_1/2_**  **(h)** |
| --- | --- | --- | --- | --- | --- | --- | --- | --- | --- | --- |
| 2 mg/kg Q2W | 61.1 | 15400 | 61.1 | 16.1 | 43.9 | 42.1 | 0.130 | 2.00 | 324 | 234 |
| 2 mg/kg Q4W | 61.1 | 15500 | 61.1 | 6.11 | 44.2 | 42.4 | 0.129 | 2.00 | 329 | 238 |
| 4 mg/kg Q2W | 122 | 30800 | 122 | 32.3 | 43.9 | 42.1 | 0.130 | 2.00 | 324 | 234 |
| 4 mg/kg Q4W | 122 | 31000 | 122 | 12.2 | 44.2 | 42.4 | 0.129 | 2.00 | 329 | 238 |
| C_eoi_ was set to simulated serum XTMAB-16 concentration at 2-hour nominal time.  Abbreviations: ADA=anti-drug antibody; AUC_inf_=area under the concentration-time curve from time 0 extrapolated to infinity; C_eoi_=end of infusion concentration; CL=clearance; C_max_=maximum concentration; C_trough_=trough concentration; MRT_inf_=mean residence time; Q2W=every 2 weeks; Q4W=every 4 weeks; T_1/2_=half-life; T_max_=time to maximum concentration; Vss=volume of distribution at steady state; Vz= apparent volume of distribution during the terminal phase | | | | | | | | | | |

## Figures

**Figure S1. In Vitro Granuloma Model of Sarcoidosis**


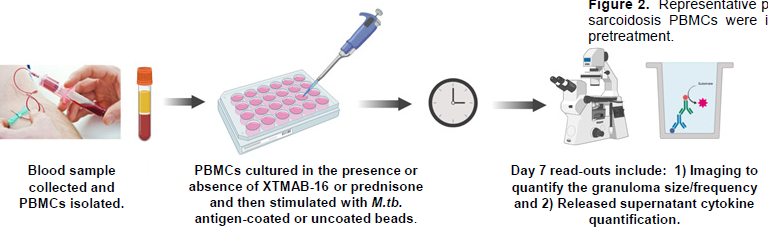


Peripheral blood mononuclear cells (PBMCs) are isolated from fresh blood using a Ficoll gradient, cultured in the presence or absence of Mycobacterium tuberculosis antigen-coated beads for 7 days, at which time granuloma formation is quantified using MIPAR image analysis (as described in the methods section). Abbreviations: PBMC = Peripheral blood mononuclear cells

**Figure S2. Representative Photomicrographs From the In Vitro Model of Sarcoidosis of Granuloma-Like Cell Aggregates**


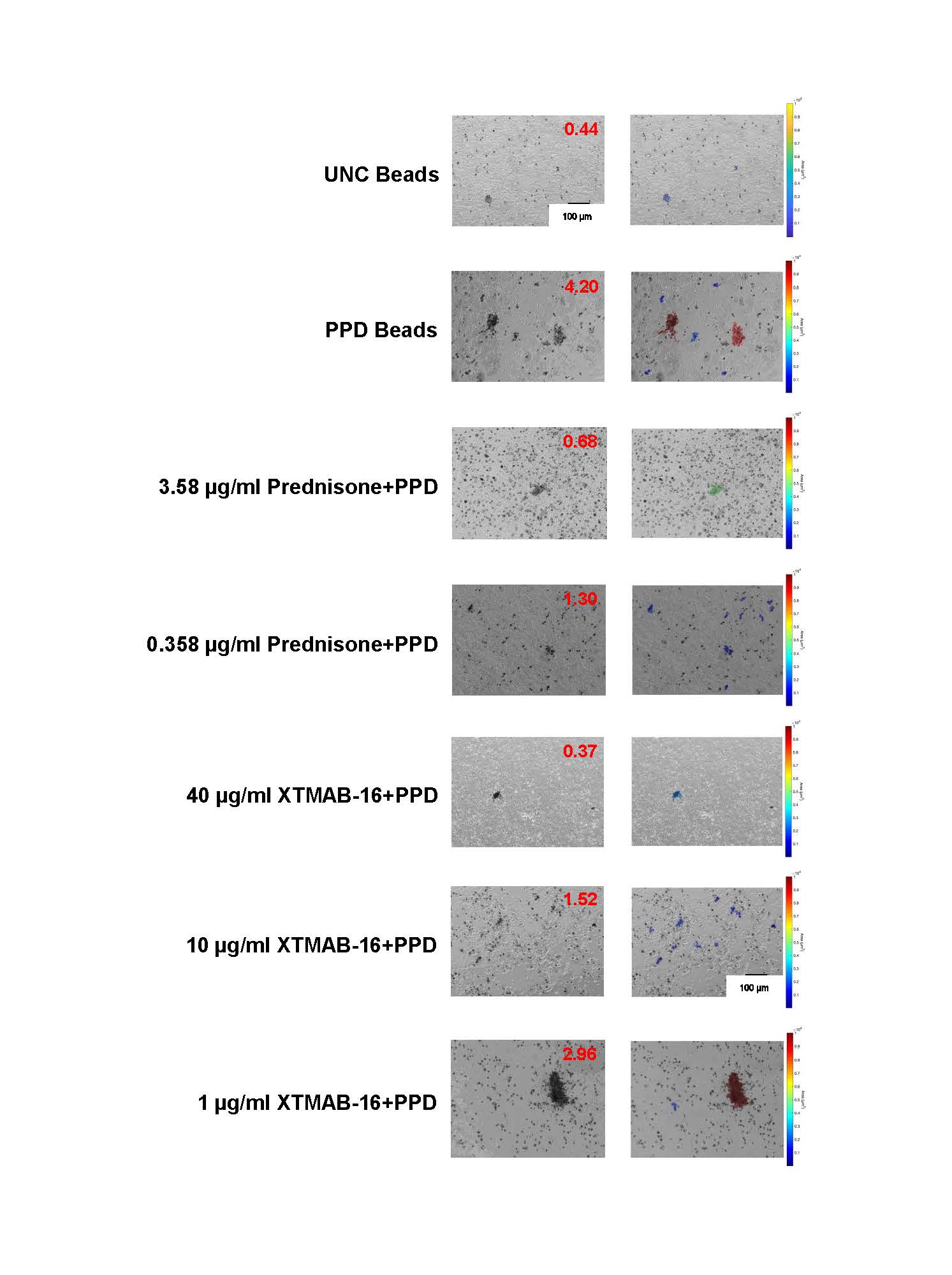


Dose dependent reduction in granuloma area percent (red inset numbers, left panels) is demonstrated in response to either prednisone or XTMAB-16. Granuloma size is further represented in the right panels based on a color spectrum wherein red represents the larger, green or blue represents smaller granulomas. Abbreviations: PPD = purified protein derivative; UNC = uncoated beads.

***Figure S3. Goodness of Fit Plots for the Final Model***


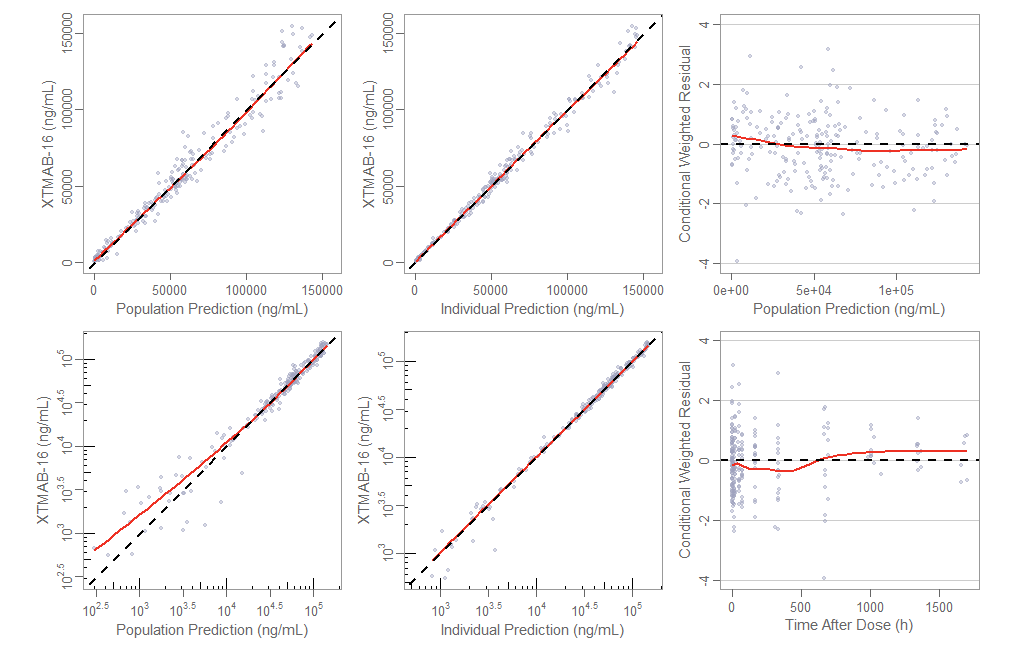


Goodness-of-fit plots suggest reasonable fit of the data. Individual- and population-predicted concentrations showed good correlation with observed data. Notes: Dots are individual data points, and solid red lines are smoothed LOESS lines. Black dashed lines show the line of unity and the 0 line in respective panels. Abbreviation: LOESS = locally weighted scatterplot smoothing.

**Figure S4. Prediction-Corrected VPC for XTMAB-16 Concentrations**


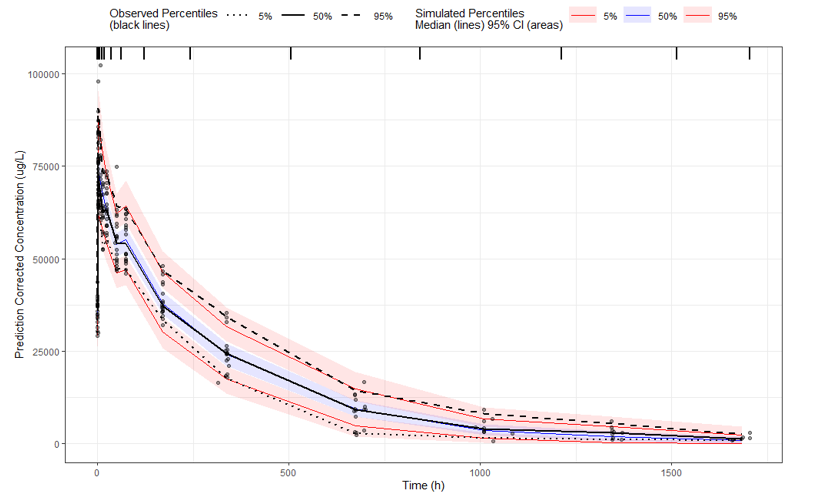


The final PPK model was able to predict the observed median and 5th and 95th percentiles of observed XTMAB-16 concentrations with good accuracy. Model evaluation by pcVPC showed suitable predictive performance of the model, which captured both the central tendency and variability in observed concentrations. Abbreviations: CL = clearance; pcVPC = predication-corrected visual predictive check.
